# People perception and stereotype-based responding: task context matters

**DOI:** 10.1007/s00426-022-01724-5

**Published:** 2022-08-22

**Authors:** Linn M. Persson, Johanna K. Falbén, Dimitra Tsamadi, C. Neil Macrae

**Affiliations:** 1 School of Psychology, University of Aberdeen, Aberdeen, AB24 3FX Scotland, UK; 2 Department of Psychology, University of Warwick, Coventry, England UK

## Abstract

Whether group impact social perception is a topic of renewed theoretical and empirical interest. In particular, it remains unclear when and how the composition of a group influences a core component of social cognition—stereotype-based responding. Accordingly, exploring this issue, here we investigated the extent to which different task requirements moderate the stereotype-related products of people perception. Following the presentation of same-sex groups that varied in facial typicality (i.e., high or low femininity/masculinity), participants had to report either the gender-related status of target words (i.e., a group-irrelevant gender-classification task) or whether the items were stereotypic or counter-stereotypic with respect to the preceding groups (i.e., a group-relevant stereotype-status task). Critically, facial typicality only impacted performance in the stereotype-status task. A further computational analysis (i.e., Diffusion Model) traced this effect to the combined operation of stimulus processing and response biases during decision-making. Specifically, evidence accumulation was faster when targets followed groups that were high (vs. low) in typicality and these arrays also triggered a stronger bias toward stereotypic (vs. counter-stereotypic) responses. Collectively, these findings elucidate when and how group variability influences people perception.

**Supplementary Information:**

The online version contains supplementary material available at 10.1007/s00426-022-01724-5.

Encountering groups is a basic facet of everyday life. Be it with classmates, friends, or co-workers, collective interactions are a regular occurrence. It is, therefore, surprising that research has generally overlooked the topic of how social groups are appraised, at least during the early stages of processing. Focusing instead on the construal of individuals, an extensive literature has explored the process and products of person perception, particularly the dynamics of stereotype-based responding (e.g., Allport, 1954; Blair, 2002; Bodenhausen & Macrae, 1998; Brewer, 1988; Fiske & Neuberg, 1990; Freeman & Ambady, 2011; Hamilton & Sherman, 1996; Kawakami et al., 2017; Kunda & Thagard, 1996; Macrae & Bodenhausen, 2000). As a result, the closely related topic of people (i.e., group) perception remains poorly understood. To redress this imbalance, recent empirical efforts have sought to identify the information that is gleaned from groups on immediate inspection (Alt & Phillips, 2022; Phillips et al., 2014). The current investigation continues in this tradition, with specific emphasis on establishing when and how group typicality influences stereotype-based responding.

## People perception and stereotyping

Processing objects and people presents the visual system with a common problem. Given fundamental attentional limitations and a world awash with highly similar items (e.g., blades of grass in a lawn, trees in a forest, faces in a crowd), how is the issue of perceptual redundancy resolved? To deal with this challenge, the mind possesses an invaluable capacity. Rather than considering every individual stimulus in intricate detail, the visual system aggregates the available group-level data and computes a statistical summary or gist (e.g., mean, variance) of a scene via a process termed ensemble coding (Alvarez, 2011; Haberman & Whitney, 2012; Whitney & Yamanashi Leib, 2018). That is, through information compression, ensemble coding enables a single representation of the shared properties of multiple objects to be derived (i.e., a group average), thereby streamlining visual processing.

Established initially for low-level object properties (e.g., size, brightness, orientation, number; Alvarez & Oliva, 2008; Ariely, 2001; Bauer, 2009; Burr & Ross, 2008; Chong et al., 2008; Dakin & Watt, 1997; Parkes et al., 2001; Watamaniuk & Duchon, 1992), comparable effects have been reported for higher-order person-related percepts, including judgments of sex, identity, and emotion (e.g., Alt et al., 2019; de Fockert & Wolfenstein, 2009; Goldenberg et al., 2020; Goodale et al., 2018; Haberman & Whitney, 2007; Neumann et al., 2013; Yang & Dunham, 2019). For example, regarding group membership, even when presented very briefly, people can readily estimate the sex-based composition of facial arrays (Yang & Dunham, 2019). Moreover, as the ratio of mixed-sex ensembles shifts to portray greater numbers of men than women, groups are judged to be more threatening and to possess increasingly sexist standards (Alt et al., 2019; Goodale et al., 2018). Additionally, in the context of emotional processing, people can extract the average emotion of groups comprising members displaying a mixture of happy and sad expressions (Haberman & Whitney, 2007).

Inspired by these findings, Persson et al. (2021) recently explored the effect that groups of varying size (i.e., 2, 3, or 4 same-sex persons) versus individuals (i.e., people vs. person perception) exert on stereotype-based responding in a sequential-priming task (Kidder et al., 2018). It was expected that, because of increased categorical intensity (Blair et al., 2005; Cassidy et al., 2017; Dixon & Maddox, 2005; Freeman & Ambady, 2009; Locke et al., 2005; Pauker & Ambady, 2009), groups (vs. individuals) would generate larger priming effects, with group size moderating the strength of stereotype-based priming. Interestingly, however, these effects were not observed. Instead, group and person primes triggered equivalent levels of stereotype-based responding (i.e., (priming was insensitive to the size of the groups). Exploring these effects further, an additional computational analysis (i.e., Diffusion Model [DM] analysis) indicated that, for both groups and individuals, stereotype-based priming was underpinned by a response bias—specifically, prime-target response compatibility—and not the enhanced processing of stereotype-related material (Falbén et al., 2019; Kidder et al., 2018; Tsamadi et al., 2020; White et al., 2018). In other words, people perception neither elevated stereotype-based responding nor entailed stereotype activation.

So why did the composition (i.e., size) of the groups fail to influence stereotype-based responding? In considering this issue, Persson et al. (2021) advanced an interesting observation. In research exploring people perception, rapidly presented visual arrays (i.e., ensembles) are always a task-relevant component of the experimental methodology. That is, to perform the task successfully, participants must report how a target stimulus relates to the previously presented array (Alvarez, 2011; Whitney & Yamamashi Leib, 2018). For example, to what extent does a test face match the mean identity (or emotional expression) portrayed in the previous ensemble (Alt et al., 2019; Goodale et al., 2018; Haberman & Whitney, 2007, 2009; Haberman et al., 2015; Yang & Dunham, 2019)? Crucially, this contrasts with priming paradigms—the dominant methodology in person perception research—in which prime-target pairings need not be considered in conjunction to generate a response (Kidder et al., 2018; Wentura & Degner, 2010). What, of course, this suggests is that, during people perception, stereotype-based responding may necessitate that attention be directed to the specific dimension of judgmental interest. In other words, only by explicitly linking the requested judgment with the previously presented group may differences in stereotype-based responding emerge (i.e., groups must be task-relevant), a possibility we explored in the current inquiry.

## The current research

To develop understanding of the process and consequences of people perception (Alt & Phillips, 2022; Phillips et al., 2014), here we considered the conditions under which variability in the composition of groups influences stereotype-based responding. Diverging from Persson et al. (2021), rather than contrasting person and people perception, in the current experiment group size was held constant and ensembles varied in facial typicality. Previous work has shown facial typicality to exert considerable influence on person construal, such that stereotype-based responding is elevated for typical [vs. atypical] exemplars (Dixon & Maddox, 2005; Freeman & Ambady, 2009; Livingston & Brewer, 2002; Locke et al., 2005; Sofer et al., 2015). Similarly, at least in task settings in which attention is directed to the composition of groups, we expected stereotype-based responding to be greater for typical (vs. atypical) ensembles.

Adopting a sequential methodology, participants were initially presented with same-sex groups (i.e., 4 women or 4 men) that varied in femininity/masculinity (i.e., high vs. low). Following the presentation of the groups, stereotyped target words (e.g., *nurse*, *perfume*, *caring*, *farmer*, *hammer*, *assertive*) were displayed and, in two different blocks, participants had to report either: (i) whether the items were feminine or masculine in implication (i.e., gender-classification task); or (ii) whether they were stereotypic or counter-stereotypic with respect to the preceding group (i.e., stereotype-status task). Thus, in one block the groups were task-irrelevant (i.e., response-priming procedure),^1^ whereas in the other block they were directly task-relevant. We expected that stereotype-based responding would be moderated by the judgmental relevance of the groups, such that groups high (vs. low) in typicality would facilitate performance, but that this effect would only emerge in the stereotype-status task. That is, facial typicality would only influence performance when the groups were task relevant (vs. irrelevant).

Of theoretical significance, an additional objective was to elucidate the cognitive pathway through which group typicality influences task performance. Accordingly, to provide this level of process specificity, data were submitted to a DM analysis (Ratcliff et al., 2016). Usefully, the DM decomposes decisional processing into the response- and stimulus-based processing operations that underpin task performance and has been applied successfully across a range of domains, including person/people construal (Falbén et al., 2019; Persson et al., 2021; Tsamadi et al., 2020). In essence, stereotype-based responding can be driven by a response bias, a stimulus processing bias, or these biases in combination (White & Poldrack, 2014). For example, following the presentation of a group, stereotypic expectancies can impact response-related operations, such that less evidence is required when selecting stereotype-consistent compared to stereotype-inconsistent responses (i.e., response bias Kidder et al., 2018; Wentura & Degner, 2010). Alternatively, and independently, through the pre-activation of associated contents in memory (Collins & Loftus, 1975), stereotype-related expectancies can influence the efficiency of visual processing, such that decisional evidence is accumulated more rapidly from stereotype-consistent than stereotype-inconsistent targets (i.e., stimulus processing bias Freeman & Ambady, 2011; Kawakami et al., 2017; Macrae & Bodenhausen, 2000).

To date, in sequential-priming tasks, stereotype-based responding has been shown to be underpinned by a response bias (Falbén et al., 2019; Persson et al., 2021; Tsamadi et al., 2020). Specifically, via a bias toward compatible (vs. incompatible) prime-target outcomes, less evidence is needed when selecting stereotype-consistent compared to stereotype-inconsistent responses, an outcome that has important implications for theoretical accounts of person perception (Kidder et al., 2018). In the DM, this effect is captured by differences in the starting point of evidence accumulation during decisional processing (White & Poldrack, 2014). Replicating this finding, we expected a similar effect to emerge when participants’ task was to report whether target words were feminine or masculine in implication (i.e., gender-classification task), a response bias that would not be impacted by the typicality of the groups. That is, although it is possible that increased categorical representativeness could trigger an elevated response bias (i.e., typical groups elicit a stronger bias toward compatible [vs. incompatible] group-target responses; Dunovan et al., 2014; White & Poldrack, 2014), we did not expect this effect to arise when the groups were irrelevant with respect to the requested judgment.

In contrast, when the task probed whether target words were stereotype-consistent or stereotype-inconsistent with respect to the preceding group (i.e., stereotype-status task), a different pattern of effects was expected to emerge. First, the evidential requirements of response selection (i.e., starting point of evidence accumulation in the DM) would be reduced when making stereotypic (i.e., group-target compatible) compared to counter-stereotypic (i.e., group-target incompatible) judgments. Second, less evidence would be required when targets followed ensembles that were high (vs. low) in typicality (Alt et al., 2019; Goodale et al., 2018). Third, as the stereotype-status (vs. gender-classification) task requires participants to compare the group and target in working memory, this should be reflected in the decisional operations that underpin task performance (Dunovan et al., 2014; White & Poldrack, 2014). In particular, groups high (vs. low) in typicality should enhance target-matching-to-template (Dixon & Maddox, 2005; Freeman & Ambady, 2009; Livingston & Brewer, 2002; Locke et al., 2005), thereby generating a stimulus processing bias. Crucially, however, rather than driving priming, decisional evidence should be extracted more rapidly when targets followed groups that were high (vs. low) in typicality and were counter-stereotypic (vs. stereotypic) in implication. Reflecting the potency of unexpected inputs, counter-stereotypic targets have been shown to enhance evidence accumulation in previous work exploring the dynamics of person construal (Falbén et al., 2019; Persson et al., 2021; Tsamadi et al., 2020). In the DM, this effect is captured by differences in the rate of information uptake during decisional processing (White & Poldrack, 2014).

Of additional interest, using a different methodology to investigate stereotype-based responding—notably, the shooter task (Correll et al., 2002)—Frenken et al. (2022) recently traced the origin of stereotype bias to differences in non-decisional processes, specifically motoric preparation (i.e., primes pre-activate stereotype-consistent motor responses, thus enhance execution readiness). Accordingly, in both the gender-classification and stereotype-status task, here we also considered the possibility that task performance may be underpinned by differences in non-decisional processing operations (Voss et al., 2013a, 2013b).

## Method

### Participants and design

Thirty-eight participants (25 female, 13 male, *M*_age_ = 23.55, *SD* = 3.94) took part in the experiment. Based on the medium effect sizes reported in both stereotype-classification and stereotype-status tasks (Falbén et al., 2019; Kidder et al., 2018), a sample of 33 participants afforded 80% power to detect an effect of *d*_*z*_ = 0.50 (PANGEA [v.0.2]). An additional ~ 15% were recruited to allow for counterbalancing and dropout. Participants were recruited using the Prolific platform for online testing (www.prolific.co), with each receiving compensation at the rate of £7.50/h. Informed consent was obtained from participants prior to the commencement of the experiment and the protocol was reviewed and approved by the Ethics Committee at the School of Psychology, University of Aberdeen. The experiment had a 2 (Ensemble: female vs. male) × 2 (Typicality: high vs. low) × 2 (Task: relevant vs. irrelevant) × 2 (Target: congruent vs. incongruent) repeated-measures design.

### Stimulus materials and procedure

Participants were informed that the study comprised word-classification tasks. In one block of trials (i.e., gender-classification task), following the presentation of single-sex groups comprising four faces, participants had to report, by the means of a key press, whether a target word was typically feminine (occupations: *receptionist, beautician, secretary, hairdresser, nurse*; objects: *perfume, doll, flower, dress, lipstick,* traits: *loyal, caring, affectionate, shy, gentle, understanding, sympathetic, warm*) or masculine (occupations: *engineer, mechanic, builder, farmer, pilot*; objects: *beer, hammer, bowtie, briefcase, cigar*, traits*: dominant, competitive, strong, decisive, commanding, athletic, assertive, ambitious*) in implication given prevailing gender stereotypes (Persson et al., 2021; Tsamadi et al., 2020). In a second block of trials (i.e., stereotype-status task), in contrast, they had to report whether, given prevailing gender stereotypes, a target word was stereotypical or counter-stereotypical with respect to the preceding group (Falbén et al., 2019; Wang et al., 2018; White et al., 2018). Importantly, the facial arrays varied in typicality, such that each contained faces that were either high or low in femininity or masculinity.

Participants performed 16 practice trials at the beginning of each block. Each block consisted of 320 experimental trials, in which stereotype-consistent (i.e., female faces/feminine item and male faces/masculine item) and stereotype-inconsistent (i.e., female faces/masculine item and male faces/feminine item) targets appeared equally often in a random order. Each trial began with the presentation of a central fixation cross for 500 ms, followed by an array comprising four female or male faces (high or low in typicality) which remained on the screen for 250 ms, after which it disappeared and was replaced by a target word (i.e., gender-typed words pertaining to objects, occupations, and traits) for 1000 ms (i.e., stimulus onset asynchrony [SOA] = 250 ms). Depending on the block, participants had to report: (i) whether the target was stereotypically feminine or masculine or (ii) stereotypical or counter-stereotypical with respect to the preceding group. Participants had 1500 ms in which to make a response and the inter-trial interval was 500 ms. The order of the blocks and the meaning of the response keys (i.e., N & M) were counterbalanced across participants.

Faces (30 female & 30 male faces) were taken from the Chicago Face Database (Ma et al., 2015), were in grayscale, depicted young Caucasian adults aged 20–30 years, and located in 2 × 2 grids that were 300 × 352 pixels in size. Of the 30 female faces, 15 were high (*M* = 5.52, *SD* = 0.14) and 15 were low (*M* = 3.38, *SD* = 0.31) in femininity (*t*(14) = 24.94, *p* < 0.001, *d*_*z*_ = 6.44). Similarly, of the 30 male faces, 15 faces were high (*M* = 5.12, *SD* = 0.18) and 15 were low (*M* = 3.51, *SD* = 0.29) in masculinity (*t*(14) = 17.44, *p* < 0.001, *d*_*z*_ = 4.50). Multiple versions of the grids were created for each condition (i.e., female-high, female-low, male-high, male-low) to ensure that faces appeared equally often in each of the locations. The to-be-judged occupations and traits were taken from Falbén et al. (2019) and the objects from Crawford et al. (2004). On completion of the experiment, participants were debriefed and thanked.

## Results

### Outlier screening

Prior to analyzing the data, participants (3 participants, 1 female) who performed at chance level were removed. Responses faster than 200 ms were excluded from the analyses, eliminating less than 1% of the overall number of trials (see Supplementary Material for a complete listing of the treatment means).

### Response time

A multilevel model analysis was used to examine the response time (RT) data. The analysis was conducted with the R package “lme4” (Pinheiro et al., 2015) and models were selected following recommendations by Matuschek et al. (2017). Using the R package “bayestestR” (Makowski et al., 2019), Bayes Factors were computed for the model comparisons (see Supplementary Materials for further details).

**Fig. 1:**
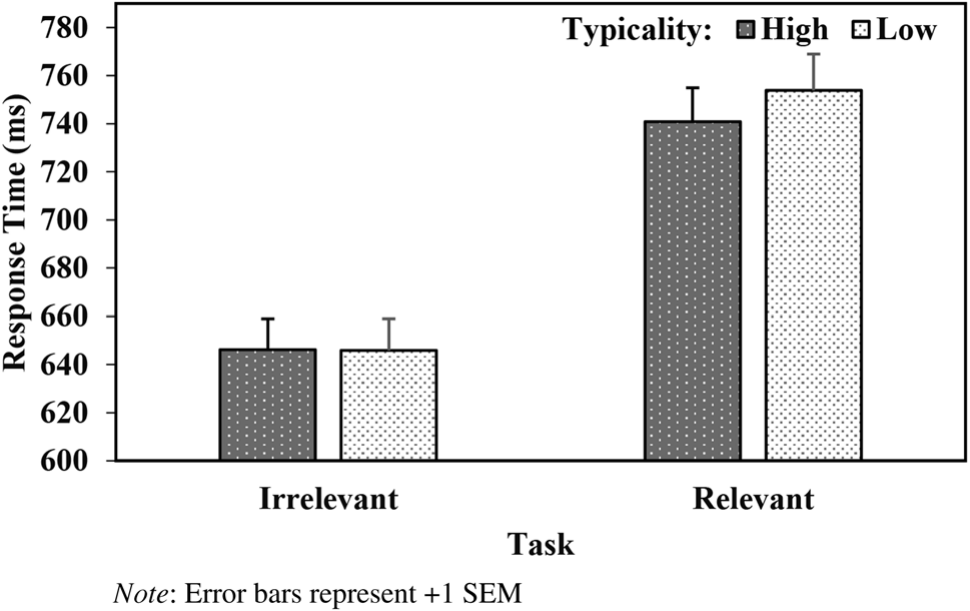
Response Time (ms) as a Function of Task and Typicality

The main effects of Ensemble, Typicality, Task, and Target and associated interactions were treated as fixed effects. Random intercepts by-participants and by-items were also included in the model, as well as random slopes for Target-by-participants. The analysis yielded main effects of Typicality (*b* = − 3.14, *SE* = 1.27, *t* = − 2.48, *p* = 0.01), Task (*b* = 50.56, *SE* = 5.98, *t* = 8.45, *p* < 0.001), and Target (*b* = − 20.37, *SE* = 1.27, *t* = − 16.07, *p* < 0.001) and a significant Ensemble X Task (*b* = -4.72, *SE* = 1.27, *t* = − 3.72, *p* < 0.001) interaction. Crucially, the analysis also revealed the important Typicality X Task (*b* = − 4.08, *SE* = 1.27, *t* = − 3.33, *p* = 0.001) interaction (see Fig. 1). On closer inspection, facial typicality only influenced performance during the group-relevant stereotype-status task (*b* = − 6.85, *SE* = 1.98, *t* = − 3.46, *p* < 0.001), such that responses were faster when targets followed ensembles that were high (vs. low) in typicality. No significant effect of typicality was observed during the group-irrelevant gender-classification task (*b* = 0.88, *SE* = 1.64, *t* = 0.54, *p* = 0.56, see Fig. 1).

**Fig. 2:**
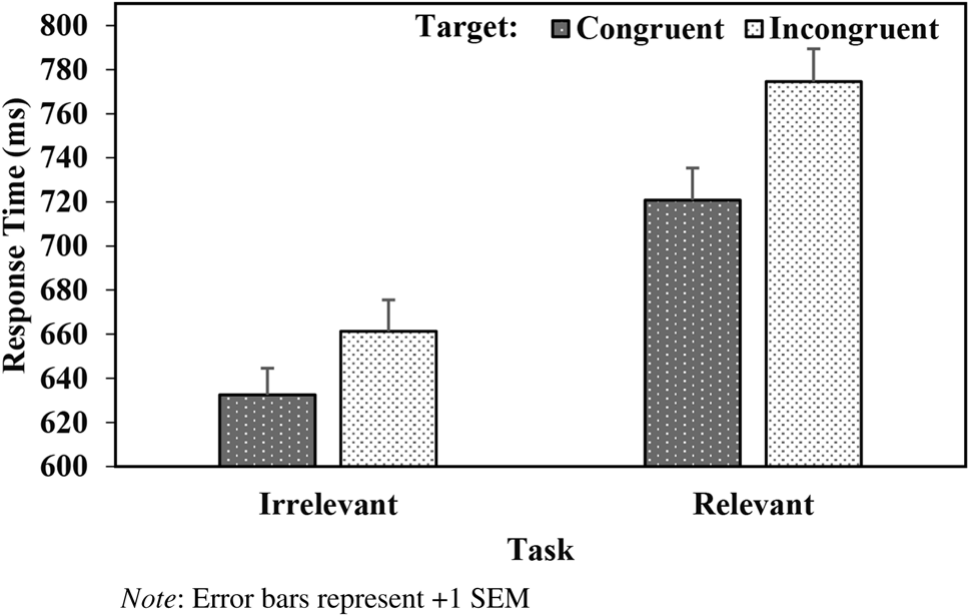
Response Time (ms) as a Function of Task and Target

The analysis also yielded a significant Task X Target (*b* = − 6.61, *SE* = 1.27, *t* = − 5.22, *p* < 0.001) interaction. Further analysis indicated that responses were faster to congruent compared to incongruent targets in both the group-irrelevant (*b* = − 23.92, *SE* = 1.64, *t* = − 8.49, *p* < 0.001) and group-relevant (*b* = − 27.10, *SE* = 1.96, *t* = − 13.83, *p* < 0.001) task (see Fig. 2).

### Accuracy

**Fig. 3:**
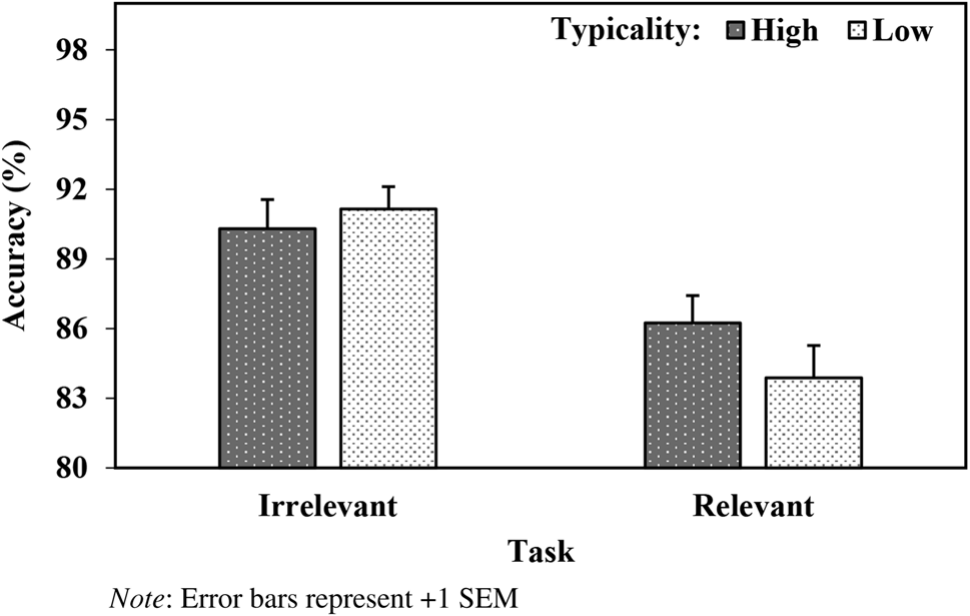
Accuracy (%) as a Function of Task and Typicality

A multilevel logistic regression analysis yielded main effects of Task (*b* = − 0.36, *SE* = 0.06, *z* = − 6.43, *p* < 0.001) and Target (*b* = 0.21, *SE* = 0.02, *z* = 9.21, *p* < 0.001). In addition, the important Task X Typicality (*b* = 0.07, *SE* = 0.02, *z* = 3.13, *p* = 0.002) interaction was observed (see Fig. 3). Further analysis revealed that, during the group-relevant stereotype-status task, responses were more accurate when targets followed ensembles that were high (vs. low) in typicality (*b* = 0.10, *SE* = 0.03, *z* = 3.66, *p* < 0.001). No significant effect of typicality was observed during the group-irrelevant gender-classification task (*b* = 0.05, *SE* = 0.03, *z* = 1.46, *p* = 0.14).

**Fig. 4:**
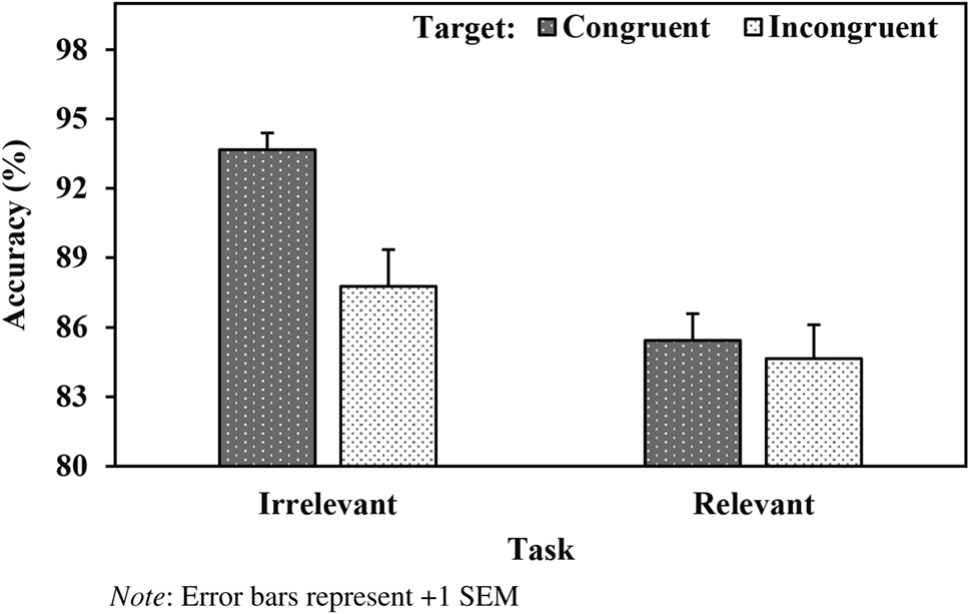
Accuracy (%) as a Function of Task and Target

The analysis also revealed a significant Task X Target interaction (*b* = − 0.17, *SE* = 0.02, *z* = − 7.63, *p* < 0.001, see Fig. 4). During the group-irrelevant task, responses were more accurate when the ensemble-target association was congruent compared to incongruent (*b* = 0.39, *SE* = 0.04, *z* = 10.76, *p* < 0.001). In contrast, no significant effect of target was observed during the group-relevant task (*b* = 0.03, *SE* = 0.03, *z* = 1.03, *p* = 0.30).

### Drift diffusion modeling

To identify the processes underpinning performance in each of the tasks, data were submitted to a DM analysis (Ratcliff et al., 2016; Voss et al., 2013a, 2013b). The benefit of this analytic approach resides in the ability of the DM to yield parameters from the RT and accuracy distributions that describe different aspects of the decision-making process (Ratcliff et al., 2016; Wagenmakers, 2009). Notably, it can reveal whether task performance was driven by stimulus processing and/or response biases. Drift rate (*v*) estimates the speed of information gathering (i.e., larger drift rate = faster evidence sampling uptake), thus is interpreted as a measure of the efficiency of stimulus processing during decision-making (i.e., stimulus bias). Boundary separation (*a*) estimates the distance between the two decision thresholds (e.g., feminine vs. masculine or stereotypic vs. counter-stereotypic), hence indicates how much evidence is required before a response is made (i.e., larger [smaller] values indicate more conservative [liberal] responding). The starting point of evidence accumulation (*z*) defines the position between the decision thresholds at which information uptake begins. If *z* is not centered between the thresholds (*z* ≠ 0.50), this denotes an a priori bias in favor of the response that is closer to the starting point (i.e., response bias). In other words, less evidence is required to reach the preferred (vs. non-preferred) threshold. Finally, the duration of all non-decisional processes is given by the additional parameter *t*_*0*_, which indicates differences in stimulus encoding and response execution.

Data were submitted to a hierarchical drift DM (HDDM) analysis (Wiecki et al., 2013). This approach assumes that the model parameters for individual participants are random samples drawn from group-level distributions and uses Bayesian statistical methods to estimate all parameters at both the group- and individual-participant level (Vandekerckhove et al., 2011). Bayesian posterior distributions were modeled using a Markov Chain Monte Carlo (MCMC) with 10,000 samples (with 5000 burn in samples). For each judgment task, eight models were estimated for comparison. First, model 1 allowed the drift rate (*v*) to vary as a function of Target (i.e., feminine vs. masculine or stereotypic vs. counter-stereotypic), Ensemble (i.e., female vs. male), and Typicality (i.e., high vs. low). The starting point (*z*) was fixed (i.e., *z* = 0.50, no bias). This model explored the possibility that task performance was underpinned exclusively by a stimulus processing bias. Model 2 was similar to Model 1, however, it also allowed the non-decisional processes *(t*_*0*_*)* to vary by Target to explore the possibility of a bias in response execution. Model 3 allowed the drift rate to vary as a function of Target and the starting point as a function of Ensemble and Typicality. This model considered whether task performance was driven solely by a response bias. Model 4 differed only in that it also allowed the non-decisional processes to vary as a function of Target. Model 5 allowed the drift rate to vary as a function of Target, Ensemble, and Typicality, and the starting point as a function of Ensemble and Typicality. As such, this model explored the possibility that task performance was underpinned by a combination of stimulus processing and response biases. Model 6 was identical to Model 5, but it also allowed the non-decisional processes to vary by Target. Model 7 allowed the drift rate to vary as a function of Target, Ensemble, and Typicality, and the starting point and non-decisional processes as a function of Ensemble and Typicality. This model explored the possibility that task performance was underpinned by a combination of stimulus processing and response biases and non-decisional processes. Lastly, Model 8 differed only in that it allowed the non-decisional processes to also vary as a function of Target. All models allowed the inter-trial variability of the non-decision (i.e., *t*_*0*_) time to vary.

Based on previous work exploring stereotype-based priming (e.g., Persson et al., 2021; Tsamadi et al., 2020), performance in the gender-classification task was expected to be underpinned by a starting point difference (i.e., *z*, response bias), such that less evidence would be required to select ensemble-compatible compared to ensemble-incompatible responses regardless of the typicality of the ensembles. In contrast, in the stereotype-status task, performance was expected to be underpinned by a combination of response and stimulus processing biases (Falbén et al., 2019). Specifically, less evidence would be required to select stereotypic compared to counter-stereotypic responses, and when targets followed ensembles that were high (vs. low) in typicality. In addition, reflecting the operation of a stimulus processing bias, information uptake would be faster when targets followed ensembles that were high (vs. low) in typicality and were incongruent (vs. congruent) in status (Falbén et al., 2019; Persson et al., 2021; Tsamadi et al., 2020).

*Gender-Classification Task.* Models were response coded, such that the upper threshold corresponded to a feminine response and the lower threshold corresponded to a masculine response (Falbén et al., 2019; Persson et al., 2021; Tsamadi et al., 2020). As can be seen from Table 1, model 4 yielded the best fit (i.e., lowest Deviance Information Criterion [DIC] value). The DIC was adopted as it is routinely used for hierarchical Bayesian model comparison (Spiegelhalter et al., 1998). As diffusion models were fit hierarchically rather than individually for each participant, a single value was calculated for each model that reflected the overall fit to the data at the participant- and group-level. Lower DIC values favor models with the highest likelihood and least number of parameters. The maximum *Ȓ* value across all parameters was 1.009, indicating that all chains converged successfully (Gelman & Rubin, 1992). To further evaluate the best fitting model, a standard model comparison procedure used in Bayesian parameter estimation—Posterior Predictive Check (PPC)—was performed (Wiecki et al., 2013). For the best fitting model, the posterior distributions of the estimated parameters were used to simulate data sets. We then assessed the quality of model fit by plotting the observed data against the simulated data for the 0.1, 0.3, 0.5, 0.7, and 0.9 RT quantiles for each experimental condition (Krypotos et al., 2015). This revealed good model fit (see Supplementary Material for the associated plots).

**Table 1:** Model Comparison (Deviance Information Criterion) for each Judgment Task

Allowed to vary by				
	Target	Ensemble	Typicality	DIC
Gender-Classification Task				
Model				
1	*v*	*v*	*V*	− 5121
2	*v, t*_*0*_	*v*	*v*	− 5176
3	*v*	*z*	*z*	− 5495
4	*v, t*_*0*_	*z*	*z*	− 5511
5	*v*	*v, z*	*v, z*	− 5355
6	*v, t*_*0*_	*v, z*	*v, z*	− 5382
7	*v*	*v, z, t*_*0*_	*v, z, t*_*0*_	− 5318
8	*v, t*_*0*_	*v, z, t*_*0*_	*v, z, t*_*0*_	− 5417

Model	Stereotype-status	Ensemble	Typicality	DIC
Stereotype-Status Task				
1	*v*	*v*	*v*	2011
2	*v, t*_*0*_	*v*	*v*	3025
3	*v*	*z*	*z*	2108
4	*v, t*_*0*_	*z*	*z*	1974
5	*v*	*v, z*	*v, z*	1795
6	*v, t*_*0*_	*v, z*	*v, z*	1646
7	*v*	*v, z, t*_*0*_	*v, z, t*_*0*_	1771
8	*v, t*_*0*_	*v, z, t*_*0*_	*v, z, t*_*0*_	1678

Note: *z* starting point, *v*  drift rate, *t*_*0*_  non-decision time

**Fig. 5:**
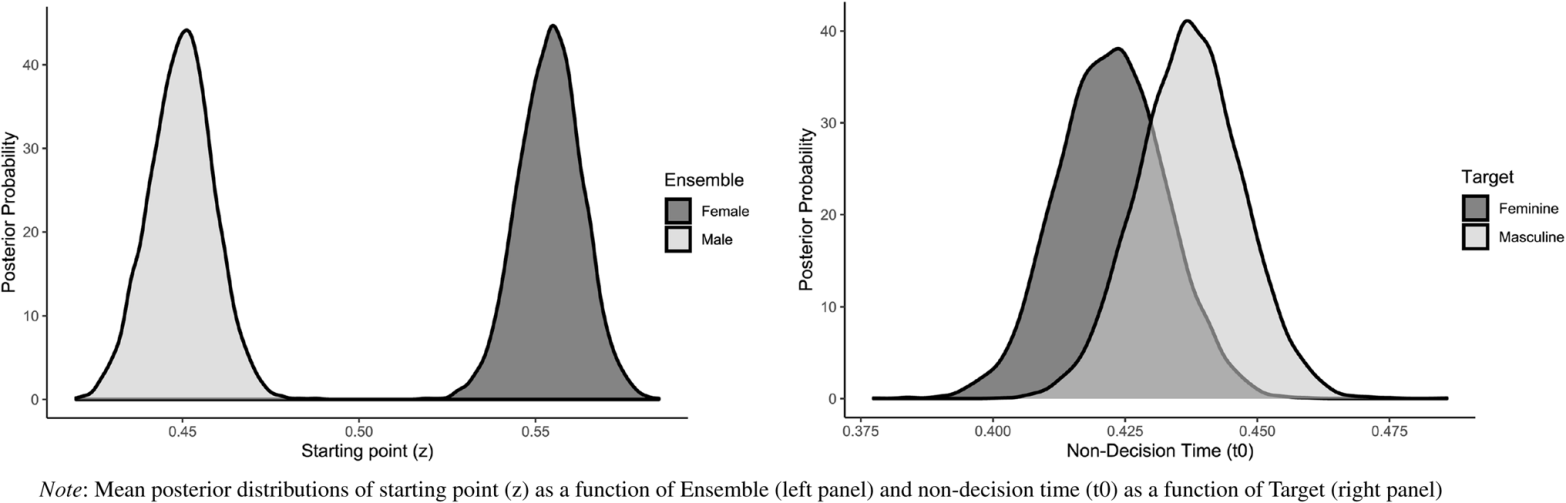
Mean Posterior Distribution Densities of the Model Parameters—Gender-Classification Task

Interrogation of the posterior distributions for the best fitting model (see Fig. 5 and Supplementary Material for parameter estimates) revealed that task performance was underpinned by biases in the evidential requirements of response selection and non-decisional processes. Consideration of the observed starting values (female-high: *z* = 0.56, female-low: *z* = 0.55, male-high: *z* = 0.44, male-low: *z* = 0.46) yielded evidence of a bias toward stereotypic (vs. counter-stereotypic) responses when feminine targets were preceded by female ensembles (*p*_Bayes_[fem-high > 0.50] < 0.001, *p*_Bayes_[fem-low > 0.50] < 0.001) and masculine target words were preceded by male ensembles (*p*_Bayes_[male-high < 0.50] < 0.001, *p*_Bayes_[male-low < 0.50] < 0.001).^2^ Facial typicality exerted no influence on the evidential requirements of response selection for either female (*p*_Bayes_[fem-high > fem-low] = 0.263) or male (*p*_Bayes_[male-high < male-low] = 0.224) ensembles. Additionally, no evidence for the operation of a stimulus processing bias was observed (*p*_Bayes_[feminine > masculine = 0.223). There was, however, a bias in non-decisional processes, such that these were faster for feminine compared to masculine targets (*p*_Bayes_[feminine < masculine] < 0.152).

**Fig. 6:**
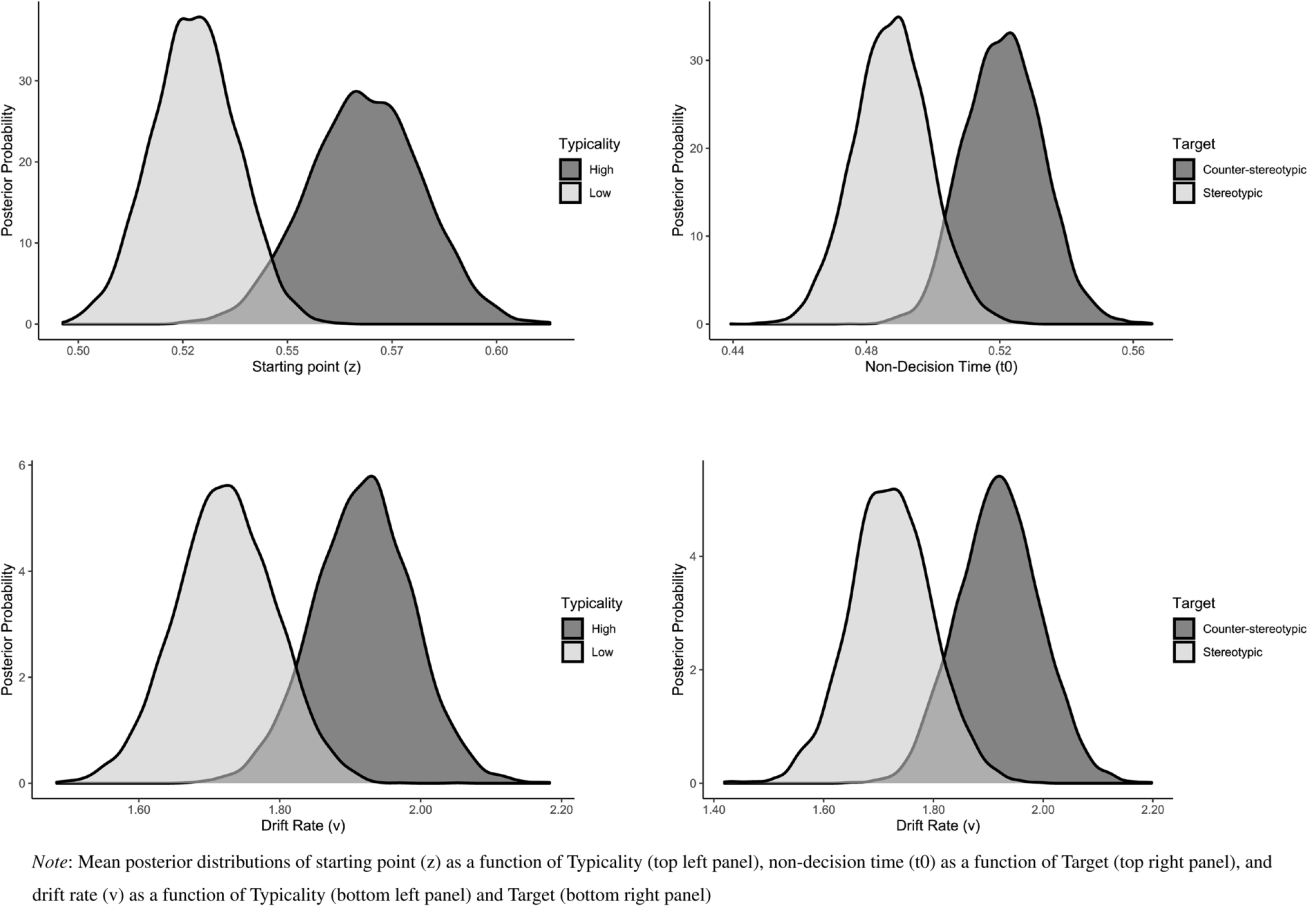
Mean Posterior Distribution Densities of the Model Parameters—Stereotype-Status Task

*Stereotype-Status Task.* Models were response coded, such that the upper threshold corresponded to a stereotypic response and the lower threshold corresponded to a counter-stereotypic response (Falbén et al., 2019). As can be seen from Table 1, model 6 yielded the best fit (lowest DIC value). The maximum *Ȓ* value across all parameters was 1.003, indicating that all chains converged successfully (Gelman & Rubin, 1992). Interrogation of the posterior distributions for the best fitting model (see Fig. 6 and Supplementary Material for parameter estimates) indicated that task performance was underpinned by a combination of response and stimulus processing biases. Consideration of the observed starting values (female-high: *z* = 0.57, female-low: *z* = 0.53, male-high: *z* = 0.52, male-low: *z* = 0.52) yielded evidence of a bias toward stereotypic (vs. counter-stereotypic) responses when targets followed both female (*p*_Bayes_[fem-high > 0.50] < 0.001, *p*_Bayes_[fem-low > 0.50] = 0.01) and male (*p*_Bayes_[male-high < 0.50] = 0.01, *p*_Bayes_[male-low < 0.50] = 0.06) ensembles. There was strong evidence that this bias toward stereotypic (vs. counter-stereotypic) responses was greater following the presentation of ensembles that were high (vs. low) in typicality (*p*_Bayes_[high > low] = 0.029). In addition, stimulus processing biases were also observed. Specifically, information uptake was faster when targets followed ensembles that were high (vs. low) in typicality (*p*_Bayes_[high > low] = 0.048) and were incongruent (vs. congruent) in implication (*p*_Bayes_[incongruent > congruent] < 0.056. Additionally, there was a bias in non-decisional processes, such that these were faster for incongruent compared to congruent targets (*p*_Bayes_[incongruent < congruent] < 0.041).

## Discussion

Extending previous work, here we demonstrated the sensitivity of people perception to the judgmental relevance of the presented groups (Persson et al., 2021). In a sequential-priming task in which prime-target pairings need not be considered to generate a response (Kidder et al., 2018; Wentura & Degner, 2010), ensemble typicality (i.e., high vs. low) failed to influence performance. Instead, a standard priming effect emerged, such that responses were faster to compatible compared to incompatible ensemble-target stimulus pairs (Persson et al., 2021; Tsamadi et al., 2020). In contrast, when judgments explicitly probed whether targets were stereotypic or counter-stereotypic with respect to the preceding ensembles (Falbén et al., 2019; Wang et al., 2018; White et al., 2018), not only did a standard priming effect emerge (i.e., compatible < incompatible), people perception was also influenced by the typicality of the facial arrays, in that responses were speeded when ensembles were high (vs. low) in typicality.

Complementing the supposed automaticity of stereotype activation during person perception (Bargh, 1999; Bodenhausen & Macrae, 1998; Brewer, 1988; Fiske & Neuberg, 1990; Freeman & Ambady, 2011; Kawakami et al., 2017; Kunda & Thagard, 1996; Macrae & Bodenhausen, 2000), comparable effects are also believed to arise during people perception. For example, in their Selection, Extraction, and Application (SEA) model, Phillips and colleagues (2014) contend that, regardless of the processing objectives in place or the availability of cognitive resources, groups that vary in composition will moderate stereotype-based responding, an effect that reflects a mandatory facet of people perception. Based on previous research, however, one may reasonably question this assumption (Whitney & Yamanashi Leib, 2018). Given that groups impact performance when participants have been instructed to compare a target stimulus with the previously presented ensemble or render a judgment on the actual ensemble itself (Alt et al., 2019; Goodale et al., 2018; Haberman & Whitney, 2007; Phillips et al., 2018; Yang & Dunham, 2019), this raises the possibility that the products of people perception may be contingent on the judgmental requirements of the immediate task setting—that is, people perception is goal dependent (Bargh, 1989; Moors & De Houwer, 2006). The current findings supported this viewpoint.

If, as has been suggested, participants are automatically sensitized to differences in the composition of groups (Phillips et al., 2014), then facial typicality should have influenced performance during the gender-classification task. As it turned out, however, this was not the case, stereotype-based responding was insensitive to the typicality of the ensembles. Indeed, only when attention was explicitly directed to ensemble-target relations did group typicality impact performance (Alvarez, 2011; Whitney & Yamanashi Leib, 2018), indicating that judgments were faster when targets were preceded by groups that were high (vs. low) in typicality. Operating in this way, people perception has the flexibility and adaptability that is required in complex social settings that are characterized by ever changing goals and task constraints. Rather than wasting time and resources processing entirely goal-irrelevant group differences, people perception can furnish this information only when it may be needed. The implications for stereotype-based responding are obvious. Countering the assumption that stereotyping is an inevitable consequence of group perception (Alt & Phillips, 2022; Phillips et al., 2014), instead it appears to be a task-dependent outcome that is likely influenced by a host of factors (e.g., processing goals, attentional resources, temporal constraints). In this way, people perception parallels the dynamics of stereotype-based responding during person perception (Blair, 2002; Macrae & Bodenhausen, 2000).

Aside from exploring the conditions under which group typicality impacts the stereotype-based products of people perception, an additional objective of the current work was to identify the processes through which these effects arise. Adopting a DM analysis (Ratcliff et al., 2016), the results revealed variability in the operations underpinning task performance across the two judgmental tasks. Replicating previous research, priming was underpinned by a response bias in the ensemble-irrelevant gender-classification task (Persson et al., 2021; Tsamadi et al., 2020), an effect that was indifferent to the typicality of the groups. In the ensemble-relevant stereotype-status task, in contrast, a combination of response and stimulus processing biases underpinned the reported effects (Falbén et al., 2019; White & Poldrack, 2014). First, a bias toward stereotypic (vs. counter-stereotypic) responses was greater when groups were high (vs. low) in typicality. Second, reflecting differences in the ease with which targets could be matched with ensembles in working memory, information uptake was faster when target items followed groups that were most representative of the category under consideration. Finally, the processing of counter-stereotypic (vs. stereotypic) material was enhanced. This effect has been reported in several recent studies exploring person perception (Falbén et al., 2019; Tsamadi et al., 2020) and reflects the informational value of expectancy-discrepant stimuli (Johnston & Hawley, 1994; Sherman et al., 1998). Collectively, these findings underscore the value of computational modeling approaches in explicating the latent processes that support people perception and its attendant stereotype-related outcomes.

Notwithstanding the potential implications of the current research for models of people perception (Alt & Phillips, 2022; Phillips et al., 2014), several important limitations must be noted. As homogenous groups (i.e., all members of the group were either high or low in typicality) were used to explore stereotype-based responding, it is possible that participants adopted a strategy whereby they focussed on only a single face in each ensemble to perform the stereotype-status task. Corroborating work in person perception, such an approach would elicit differences between individuals high versus low in facial typicality (Falbén et al., 2019). To eliminate this possibility, heterogenous groups should be presented where members all vary on the specific dimension (e.g., femininity/masculinity) of interest (Bucher & Voss, 2019; Lerche et al., 2019). Alternatively, adopting the current methodology, a group with four members could be contrasted with a single person. Extending Persson et al. (2021), one would expect a group (vs. individual) to increase stereotype-based responding during the stereotype-status task. Finally, the reported modeling results reflect basic differences in the cognitive operations required to perform the gender-classification and stereotype-status tasks, respectively. Notably, whereas the stereotype-status task required explicit comparison of the group and target in working memory, the gender-classification task did not. In elucidating the cognitive processes that underpin people perception, an alternative (and better) strategy would be to manipulate the processing of the group but using a common judgment task (e.g., Macrae et al., 1997; Wheeler & Fiske, 2005).

Moving forward, future research should consider how other group-related characteristics influence people perception. An oft reported finding in the person perception literature is that first impressions of faces are computed quickly and effortlessly along a set of fundamental dimensions; including trustworthiness, competence, dominance, and intelligence (e.g., Oosterhof & Todorov, 2008; Sutherland et al., 2013; Todorov et al., 2015; Wills & Todorov, 2006; Zebrowitz, 2017). What has yet to attract empirical attention, however, is how groups that vary in terms of these characteristics (e.g., high vs. low trustworthiness) influence people perception. Based on the current findings, it may be tempting to conclude that groups only influence responding when they are directly goal (i.e., judgment) relevant to perceivers. In contrast, if as has been argued, these impressions are primary, adaptive, and culturally universal because of their signal value (e.g., Schaller, 2008; Todorov et al., 2015; Zebrowitz, 2004), it is possible that mere exposure to groups may be sufficient to elicit the corresponding inferences. For example, when groups convey information with immediate and important implications (e.g., threat, danger), group variability may influence decision-making without instruction.

## Conclusion

Using different judgment tasks (i.e., gender-classification vs. stereotype-status), here we demonstrated when and how group typicality influences stereotype-based responding. Most notably, group typicality only moderated performance when attention was explicitly directed to group-target relations (i.e., stereotype-status task), such that responses were speeded when facial arrays were high (vs. low) in typicality. Absent this requirement (i.e., gender-classification task), group typicality exerted no influence on performance. Extending these findings, an additional DM analysis demonstrated that group typicality impacted performance in the stereotype-status task through a combination of response (i.e., evidential requirements of response selection) and stimulus processing (i.e., speed of information uptake) biases. At least in the context of stereotype-based responding, these findings inform understanding of the process and products of people perception.

## Supplementary Information

Below is the link to the electronic supplementary material.

Supplementary file1 (DOCX 140 KB)

## Data Availability

Available on request from the first author.

## Footnotes

1 Meta-analytic work has revealed robust stereotype-based priming effects when response-priming tasks have been used, but non-significant effects when semantic-priming tasks have been adopted (Kidder et al., 2018). As such, a response-priming task was employed in the current experiment.

2 Bayesian *p* values quantify the degree to which the difference in the posterior distribution is consistent with the hypothesis that the parameter is greater for consistent than inconsistent responses. For example, a Bayesian *p* of .05 indicates that 95% of the posterior distribution supports the hypothesis (Kruschke, 2010; Marsman & Wagenmakers, 2017).

## Code availability

Available on request from first author.

## References

[CR1] Allport GW (1954). The nature of prejudice.

[CR2] Alt NP, Phillips LT (2022). Person perception, meet people perception: Exploring the social vision of groups. Perspectives on Psychological Science.

[CR3] Alt NP, Goodale B, Lick DJ, Johnson KL (2019). Threat in the company of men: Ensemble perception and threat evaluations of groups varying in sex ratio. Social Psychological and Personality Science.

[CR4] Alvarez GA (2011). Representing multiple objects as an ensemble enhances visual cognition. Trends in Cognitive Sciences.

[CR5] Alvarez GA, Oliva A (2008). The representation of simple ensemble visual features outside the focus of attention. Psychological Science.

[CR6] Ariely D (2001). Seeing sets: Representation by statistical properties. Psychological Science.

[CR7] Bargh JA, Uleman JS, Bargh JA (1989). Conditional automaticity: Varieties of automatic influence in social perception and cognition. Unintended thought.

[CR8] Bargh JA, Chaiken S, Trope Y (1999). The cognitive monster: The case against the controllability of automatic stereotype effects. Dual-process theories in social psychology.

[CR9] Bauer B (2009). Does Steven’s *power law for brightness extend to perceptual brightness averaging?*. Psychological Research Psychologische Forschung.

[CR10] Blair IV (2002). The malleability of automatic stereotypes and prejudice. Personality and Social Psychology Review.

[CR11] Blair IV, Chapleau KM, Judd CM (2005). The use of Afrocentric features as cues for judgment in the presence of diagnostic information. European Journal of Social Psychology.

[CR12] Bodenhausen GV, Macrae CN (1998). Stereotype activation and inhibition. Advances in Social Cognition.

[CR13] Brewer MB (1988). A dual process model of impression formation. Advances in Social Cognition.

[CR14] Bucher A, Voss A (2019). Judging the mood of the crowd: Attention is focused on happy faces. Emotion.

[CR15] Burr D, Ross J (2008). A visual sense of number. Current Biology.

[CR16] Cassidy BS, Sprout GT, Freeman JB, Krendl AC (2017). Looking the part (to me): Effects of racial prototypicality on race perception vary by prejudice. Social Cognitive and Affective Neuroscience.

[CR17] Chong SC, Joo SJ, Emmmanouil TA, Treisman A (2008). Statistical processing: Not so implausible after all. Perception & Psychophysics.

[CR18] Collins AM, Loftus EF (1975). A spreading-activation theory of semantic processing. Psychological Review.

[CR19] Correll J, Park B, Judd CM, Wittenbrink B (2002). The police officer’s dilemma: Using ethnicity to disambiguate potentially threatening individuals. Journal of Personality and Social Psychology.

[CR20] Crawford JT, Leynes PA, Mayhorn CB, Bink ML (2004). Champagne, beer, or coffee? A corpus of gender-related and neutral words. Behavior Research Methods, Instruments & Computers.

[CR21] Dakin SC, Watt RJ (1997). The computation of orientation statistics from visual texture. Vision Research.

[CR22] De Fockert JW, Wolfenstein C (2009). Rapid extraction of mean identity from sets of faces. Quarterly Journal of Psychology.

[CR23] Dixon TL, Maddox KB (2005). Skin tone, crime news, and social reality judgments: Priming the stereotype of the dark and dangerous black criminal. Journal of Applied Social Psychology.

[CR24] Dunovan KE, Tremel JJ, Wheeler ME (2014). Prior probability and feature predictability interactively bias perceptual decisions. Neuropsychologia.

[CR25] Falbén JK, Tsamadi D, Golubickis M, Olivier JL, Persson LM, Cunningham WA, Macrae CN (2019). Predictably confirmatory: The influence of stereotypes during decisional processing. Quarterly Journal of Experimental Psychology.

[CR26] Fiske ST, Neuberg SL (1990). A continuum of impression formation, from category-based to individuating processes: Influences of information and motivation on attention and interpretation. Advances in Experimental Social Psychology.

[CR27] Freeman JB, Ambady N (2009). Motions of the hand expose the partial and parallel activation of stereotypes. Psychological Science.

[CR28] Freeman JB, Ambady N (2011). A dynamic interactive theory of person construal. Psychological Review.

[CR29] Frenken M, Hemmerich W, Izydorczyk D, Scharf S, Imhoff R (2022). Cognitive processes behind the shooter bias: Dissecting response bias, motor preparation, and information accumulation. Journal of Experimental Social Psychology.

[CR30] Gelman A, Rubin DB (1992). Inference from iterative simulation using multiple sequences. Statistical Science.

[CR31] Goldenberg A, Sweeny TD, Shpigel E, Gross JJ (2020). Is this my group or not? The role of ensemble coding of emotional expressions in group categorization. Journal of Experimental Psychology: General.

[CR32] Goodale BM, Alt NP, Lick DJ, Johnson KL (2018). Groups at a glance: Perceivers infer social belonging in a group based on perceptual summaries of sex ratio. Journal of Experimental Psychology: General.

[CR33] Haberman J, Lee P, Whitney D (2015). Mixed emotions: Sensitivity to facial variance in a crowd of faces. Journal of Vision.

[CR34] Haberman J, Whitney D (2007). Rapid extraction of mean emotion and gender from sets of faces. Current Biology.

[CR35] Haberman J, Whitney D (2009). Seeing the mean: Ensemble coding for sets of faces. Journal of Experimental Psychology: Human Perception and Performance.

[CR36] Haberman, J., & Whitney, D. (2012). Ensemble perception: Summarizing the scene and broadening the limits of visual processing. *From perception to consciousness: Searching with Anne Treisman*, 339–349.

[CR37] Hamilton DL, Sherman SJ (1996). Perceiving persons and groups. Psychological Review.

[CR38] Johnston WA, Hawley KJ (1994). Perceptual inhibition of expected inputs: The key that opens closed minds. Psychonomic Bulletin & Review.

[CR39] Kawakami K, Amodio DM, Hugenberg K (2017). Intergroup perception and cognition: An integrative framework for understanding the causes and consequences of social categorization. Advances in Experimental Social Psychology.

[CR40] Kidder CK, White KR, Hinojos MR, Sandoval M, Crites SL (2018). Sequential stereotype priming: A meta-analysis. Personality and Social Psychology Review.

[CR41] Kruschke JK (2010). Bayesian data analysis. Wires Cognitive Science.

[CR42] Krypotos A-M, Beckers T, Kindt M, Wagenmakers, E.- J.  (2015). A Bayesian hierarchical diffusion model decomposition of performance in approach-avoidance tasks. Cognition and Emotion.

[CR43] Kunda Z, Thagard P (1996). Forming impressions from stereotypes, traits, and behaviors: A parallel-constraint-satisfaction theory. Psychological Review.

[CR44] Lerche V, Christmann U, Voss A (2019). Impact of context information on metaphor elaboration. Experimental Psychology.

[CR45] Livingston RW, Brewer MB (2002). What are we really priming? Cue-based versus category-based processing of facial stimuli. Journal of Personality and Social Psychology.

[CR46] Locke V, Macrae CN, Eaton JL (2005). Is person categorization modulated by exemplar typicality?. Social Cognition.

[CR47] Ma DS, Correll J, Wittenbrink B (2015). The Chicago face database: A free stimulus set of faces and norming data. Behavior Research Methods.

[CR48] Macrae CN, Bodenhausen GV (2000). Social cognition: Thinking categorically about others. Annual Review of Psychology.

[CR49] Macrae CN, Bodenhausen GV, Milne AB, Thorn TM, Castelli L (1997). On the activation of social stereotypes: The moderating role of processing objectives. Journal of Experimental Social Psychology.

[CR50] Makowski D, Ben-Shachar MS, Lüdecke D (2019). bayestestR: Describing effects and their uncertainty, existence, and significance within the Bayesian framework. Journal of Open Source Software.

[CR51] Marsman M, Wagonmakers E-J (2017). Three insights from a Bayesian interpretation of the one-sided *p* value. Educational and Psychological Measurement.

[CR52] Matuschek H, Kliegl R, Vasishth S, Baayen H, Bates D (2017). Balancing Type 1 error and power in linear mixed models. Journal of Memory and Language.

[CR53] Moors A, De Houwer J (2006). Automaticity: A theoretical and conceptual analysis. Psychological Bulletin.

[CR54] Neumann MF, Schweinberger SR, Burton AM (2013). Viewers extract mean and individual identity from sets of famous faces. Cognition.

[CR55] Oosterhof NN, Todorov A (2008). The functional basis of face evaluation. Proceedings of the National Academy of Sciences.

[CR56] Parkes L, Lund J, Angelucci A (2001). Compulsory averaging of crowded orientation signals in human vision. Nature Neuroscience.

[CR57] Pauker K, Ambady N (2009). Multiracial faces: How categorization affects memory at the boundaries of race. Journal of Social Issues.

[CR58] Persson LM, Golubickis M, Dublas D, Mastnak N, Falbén JK, Tsamadi D, Caughey S, Svensson S, Macrae CN (2021). Comparing person and people perception: Multiple group members do not increase stereotype priming. Quarterly Journal of Experimental Psychology.

[CR59] Phillips LT, Slepian ML, Hughes BL (2018). Perceiving groups: The people perception of diversity and hierarchy. Journal of Personality and Social Psychology.

[CR60] Phillips LT, Weisbuch M, Ambady N (2014). People perception: Social vision of group and consequences for organizing and interacting. Research in Organizational Behavior.

[CR61] Pinheiro, J., Bates, D., DebRoy, S., Sarkar, D., & R Development Core Team. (2015). *nlme: Linear and nonlinear mixed effects models*. The Comprehensive R Archive Network (CRAN), Vienna, Austria.

[CR62] Ratcliff R, Smith PL, Brown SD, McKoon G (2016). Diffusion decision model: Current issues and history. Trends in Cognitive Sciences.

[CR63] Schaller M, Ambady N, Skowronski JJ (2008). Evolutionary bases of first impressions. First impressions.

[CR64] Sherman JW, Lee AY, Bessenoff GR, Frost LA (1998). Stereotype efficiency reconsidered: Encoding flexibility under cognitive load. Journal of Personality and Social Psychology.

[CR65] Sofer C, Dotsch R, Wigboldus DH, Todorov A (2015). What is typical is good: The influence of face typicality on perceived trustworthiness. Psychological Science.

[CR66] Spiegelhalter, D. J., Best, N. G., Carlin, B. P., & van der Linde, A. (1998). *Bayesian deviance, the effective number of parameters, and the comparison of arbitrarily complex models*. Research Report, pp. 98–1009.

[CR67] Sutherland CA, Oldmeadow JA, Santos IM, Towler J, Burt DM, Young AW (2013). Social inferences from faces: Ambient images generate a three-dimensional model. Cognition.

[CR68] Todorov A, Olivola CY, Dotsch R, Mende-Siedlecki P (2015). Social attributions from faces: Determinants, consequences, accuracy, and functional significance. Annual Review of Psychology.

[CR69] Tsamadi D, Falbén JK, Persson LM, Golubickis M, Caughey S, Sahin B, Macrae CN (2020). Stereotype-based priming without stereotype activation: A tale of two priming tasks. Quarterly Journal of Experimental Psychology.

[CR70] Vanderkerckhove J, Tuerlinckx F, Lee MD (2011). Hierarchical diffusion models for two-choice response times. Psychological Methods.

[CR71] Voss A, Nagler M, Lerche V (2013). Diffusion models in experimental psychology. Experimental Psychology.

[CR72] Voss A, Rothermund K, Gast A, Wentura D (2013). Cognitive processes in associative and categorical priming: A diffusion model analysis. Journal of Experimental Psychology: General.

[CR73] Wagenmakers E-J (2009). Methodological and empirical developments for the Ratcliff diffusion model of response times and accuracy. European Journal of Cognitive Psychology.

[CR74] Wang P, Tan C-H, Zhang YLQ, Wang Y-B, Luo J-L (2018). Event-related potential N270 as an index of social information conflict in explicit processing. International Journal of Psychophysiology.

[CR75] Watamaniuk SN, Ducon A (1992). The human visual system averages speed information. Vision Research.

[CR76] Wentura D, Degner J, Gawronski B, Payne BK (2010). A practical guide to sequential priming and related tasks. Handbook of implicit social cognition: Measurement, theory, and applications.

[CR77] Wheeler ME, Fiske ST (2005). Controlling racial prejudice: Social-cognitive goals affect amygdala and stereotype activation. Psychological Science.

[CR78] White CN, Poldrack RA (2014). Decomposing bias in different types of simple decisions. Journal of Experimental Psychology: Learning, Memory, and Cognition.

[CR79] White KRG, Danek RH, Herring DR, Taylor JH, Crites SL (2018). Taking priming to task: Variations in stereotype priming effects across participant task. Social Psychology.

[CR80] Whitney D, Yamanashi Leib A (2018). Ensemble perception. Annual Review of Psychology.

[CR81] Wiecki TV, Sofer I, Frank MJ (2013). HDDM: Hierarchical Bayesian estimation of the drift-diffusion model in Python. Frontiers in Neuroinformatics.

[CR82] Willis J, Todorov A (2006). First impressions: Making up your mind after a 100-ms exposure to a face. Psychological Science.

[CR83] Yang X, Dunham Y (2019). Hard to disrupt: Categorization and enumeration by gender and race from mixed displays. Journal of Experimental Social Psychology.

[CR84] Zebrowitz LA (2004). The origin of first impressions. Journal of Cultural and Evolutionary Psychology.

[CR85] Zebrowitz LA (2017). First impressions from faces. Current Directions in Psychological Science.

