# Supplementary material for: People perception and stereotype-based responding: task context matters
Source: Psychol Res. 2022 Aug 22;87(4):1219–31. doi: 10.1007/s00426-022-01724-5 (PMC10191924; doi:10.1007/s00426-022-01724-5)
Supplement: Supplementary file 1 — Supplementary file1 (DOCX 140 KB) [file 426_2022_1724_MOESM1_ESM.docx]

**Title:** People Perception and Stereotype-Based Responding: Task Context Matters

**Journal:** Psychological Research

# Authors: Linn M. Persson, Johanna K. Falbén, Dimitra Tsamadi, C. Neil Macrae

**Corresponding Author:** Linn Persson, University of Aberdeen, l.persson.19@abdn.ac.uk

**Supplementary Material**

Combined Table of Means

*Table S1.* Reaction Time (ms) and Accuracy (%) as a Function of Task, Ensemble, Typicality, and Target.

Task Ensemble Typicality Target Response Time (ms) Accuracy (%)

Stereotype-Status

female high congruent 706 (97) 89 (11)

incongruent 764 (83) 85 (11)

low congruent 715 (100) 86 (13)

incongruent 783 (92) 84 (11)

male high congruent 735 (106) 84 (12)

incongruent 771 (100) 86 (9)

low congruent 746 (102) 82 (14)

incongruent 784 (107) 83 (13)

Gender-Classification

female high congruent 618 (77) 94 (9)

incongruent 678 (88) 86 (13)

low congruent 623 (74) 93 (6)

incongruent 673 (92) 88 (12)

male high congruent 645 (84) 94 (4)

incongruent 649 (77) 88 (11)

low congruent 646 (78) 94 (5)

incongruent 648 (95) 90 (10)

Note. Standard deviations (*SD*) appear within parentheses.

*Table S2.* Model comparison using Bayes Factor for Response Time.

Model BF_against model 1_ BF_against model 5_

1.task*ensemble*typicality*target + (1|participant)+(1|item) 1.00 1/(2.42*10^137^)

2.task*ensemble*typicality*target + (1+task|participant)+(1|item) 5.50*10^127^ 1/(4.41*10^09^)

3.task*ensemble*typicality*target + (1+ensemble|participant)+(1|item) 8.18*10^09^ 1/(2.96*10^137^)

4.task*ensemble*typicality*target + (1+target |participant)+(1|item) 1/(3.63) 1/(8.80*10^137^)

5.task*ensemble*typicality*target + (1+ensemble+task |participant)+(1|item) 2.42*10^137^  1

6.task*ensemble*typicality*target + (1+target+task |participant)+(1|item) 6.35*10^125^  1/(3.81*10^11^)

7.task*ensemble*typicality*target + (1+target+task+ensemble |participant)+(1|item) 2.35*10^133^  1/(1.03*10^04^)

Note. Against denominator (model 1), Bayes factors were computed using the BIC approximation, by which BF10 = exp((BIC0 - BIC1)/2). Compared to model 1, we found extreme evidence in favour of model 2; extreme evidence in favour of model 2; moderate evidence against model 4 (the least supported model); extreme evidence in favour of model 5 (the most supported model); extreme evidence in favour of model 6; extreme evidence in favour of model 7 (Jeffreys, 1961).

*Table S3.* Model comparison using Bayes Factor for Accuracy.

Model BF_against model 1_ BF_against model 5_

1.task*ensemble*typicality*target + (1|participant)+(1|item) 1.00 1/(5.26*10^35^)

2.task*ensemble*typicality*target + (1+task|participant)+(1|item) 8.77*10^20^ 1/(5.99*10^14^)

3.task*ensemble*typicality*target + (1+ensemble|participant)+(1|item) 1.03*10^17^ 1/(5.12*10^18^)

4.task*ensemble*typicality*target + (1+target |participant)+(1|item) 2.01*10^03^ 1/(2.62*10^32^)

5.task*ensemble*typicality*target + (1+ensemble+task |participant)+(1|item) 5.26*10^35^  1

6.task*ensemble*typicality*target + (1+target+task |participant)+(1|item) 7.79*10^20^ 1/(6.75*10^14^)

7.task*ensemble*typicality*target + (1+target+task+ensemble |participant)+(1|item) 2.42*10^33^  1/(217.31)

Note. Compared to model 1 (the least supported model), we found extreme evidence in favour of model 2; extreme evidence in favour of model 3; extreme evidence in favour of model 4; extreme evidence in favour of model 5 (the most supported model); extreme evidence in favour of model 6; extreme evidence in favour of model 7.

*Table S4.* Deviance information criterion (DIC) for each model (Stereotype-Status Task).

Model Target Ensemble Typicality DIC

1. *v* *v* *v* 2011

2. *v, t_0_* *v* *v* 3025

3. *v* *z* *z* 2108

4. *v, t_0_* *z*  *z*  1974

5. *v* *v,z* *v,z* 1795

6. *v, t_0_* *v,z*  *v,z*  1646

7. *v* *v,z,t_0_* *v,z,t_0_* 1771

8. *v, t_0_* *v,z,t_0_*  *v,z,t_0_*  1678

Note. *v* = drift rate, *z* = starting point. *t_0_* = non-decision time. A DIC difference of 10 is strong evidence for a model.

*Table S5.* Deviance information criterion (DIC) for each model (Gender-Classification Task).

Model Target Ensemble Typicality DIC

1. *v* *v* *v* -5121

2. *v, t_0_* *v* *v* -5176

3. *v* *z*  *z*  -5495

4. *v, t_0_* *z*  *z*  -5511

5. *v* *v,z*  *v,z*   *-*5355

6 *v, t_0_* *v,z*  *v,z*  -5382

7. *v* *v,z,t_0_*  *v,z,t_0_*  -5318

8. *v, t_0_* *v,z,t_0_*  *v,z,t_0_*  -5417

Note. *v* = drift rate, *z* = starting point, *t_0_* = non-decision time. A DIC difference of 10 is strong evidence for a model.

*Table S6*. Parameter means and the upper (97.5q) and lower (2.5q) quantiles of the best fitting model (Stereotype-Status Task).

Quantile

Diffusion Model Parameter Mean 2.5q 97.5q

*a* 1.146 1.080 1.215

*v_stereo female high_* 1.873 1.584 2.170

*v_stereo female low_* 1.880 1.581 2.179

*v_stereo male high_* 1.623 1.340 1.910

*v_stereo male low_* 1.512 1.235 1.798

*v_counter female high_* -2.269 -2.654 -1.972

*v_counter female low_* -1.809 -2.097 -1.530

*v_counter male high_* -1.909 -2.208 -1.622

*v_counter male low_* -1.690 -1.972 -1.411

*t_0counter-stereotypic_* 0.521 0.500 0.544

*t_0stereotypic_* 0.488 0.465 0.510

*z_female high_* 0.569 0.542 0.595

*z_female low_* 0.535 0.507 0.562

*z_male high_* 0.521 0.494 0.548

*z_male low_* 0.521 0.495 0.546

Note. *a* = threshold separation, *v* = drift rate, *t_0_* = non-decision time, *z* = starting point.

*Table S7*. Parameter means and the upper (97.5q) and lower (2.5q) quantiles of the best fitting model (Gender-Classification Task).

Quantile

Diffusion Model Parameter Mean 2.5q 97.5q

*a* 1.157 1.084 1.238

*v_feminine_* 2.470 2.208 2.729

*v_masculine_ -*2.334 -2.604 -2.077

*t_0feminine_* 0.422 0.403 0.442

*t_0masculine_* 0.437 0.418 0.457

*z_female high_* 0.559 0.536 0.581

*z_female low_* 0.550 0.528 0.571

*z_male high_* 0.444 0.422 0.465

*z_male low_* 0.455 0.433 0.478

Note. *a* = threshold separation, *v* = drift rate*t_0_* = non-decision time, *z* = starting point.

*Figure S1*. Posterior Predictive Check. Comparison of simulated data generated by the best fitting model (i.e., model 3) and observed data for each experimental condition for the .1, .3, .5, .7, and .9 RT quantiles – Stereotype-Status Task.

*Figure S2*. Posterior Predictive Check. Comparison of simulated data generated by the best fitting model (i.e., model 2) and observed data for each experimental condition for the .1, .3, .5, .7, and .9 RT quantiles – Gender-Classification Task.

**References**

Jeffreys, H. (1961). *Theory of Probability*, Ed. 3 Oxford University Press.
